# Estimating the transmission advantage of the D614G mutant strain of SARS-CoV-2, December 2019 to June 2020

**DOI:** 10.2807/1560-7917.ES.2021.26.49.2002005

**Published:** 2021-12-09

**Authors:** Kathy Leung, Yao Pei, Gabriel M Leung, Tommy TY Lam, Joseph T Wu

**Affiliations:** 1 WHO Collaborating Centre for Infectious Disease Epidemiology and Control, School of Public Health, LKS Faculty of Medicine, The University of Hong Kong, Hong Kong SAR, China; 2 Laboratory of Data Discovery for Health (D^2^4H), Hong Kong Science Park, Hong Kong SAR, China; 3 State Key Laboratory of Emerging Infectious Diseases, School of Public Health, The University of Hong Kong, Hong Kong SAR, China; 4 Joint Institute of Virology (Shantou University and The University of Hong Kong), Guangdong-Hongkong Joint Laboratory of Emerging Infectious Diseases, Shantou University, Shantou, China

**Keywords:** D614G, SARS-CoV-2, COVID-19, fitness, mutation, variant of concern, phylogenetics, transmission advantage

## Abstract

**Introduction:**

The SARS-CoV-2 lineages carrying the amino acid change D614G have become the dominant variants in the global COVID-19 pandemic. By June 2021, all the emerging variants of concern carried the D614G mutation. The rapid spread of the G614 mutant suggests that it may have a transmission advantage over the D614 wildtype.

**Aim:**

Our objective was to estimate the transmission advantage of D614G by integrating phylogenetic and epidemiological analysis.

**Methods:**

We assume that the mutation D614G was the only site of interest which characterised the two cocirculating virus strains by June 2020, but their differential transmissibility might be attributable to a combination of D614G and other mutations. We define the fitness of G614 as the ratio of the basic reproduction number of the strain with G614 to the strain with D614 and applied an epidemiological framework for fitness inference to analyse SARS-CoV-2 surveillance and sequence data.

**Results:**

Using this framework, we estimated that the G614 mutant is 31% (95% credible interval: 28–34) more transmissible than the D614 wildtype. Therefore, interventions that were previously effective in containing or mitigating the D614 wildtype (e.g. in China, Vietnam and Thailand) may be less effective against the G614 mutant.

**Conclusion:**

Our framework can be readily integrated into current SARS-CoV-2 surveillance to monitor the emergence and fitness of mutant strains such that pandemic surveillance, disease control and development of treatment and vaccines can be adjusted dynamically.

## Introduction

Recent studies of severe acute respiratory syndrome coronavirus 2 (SARS-CoV-2) genomes have identified various mutations associated with different emerging genetic clades. Two major clades were initially reported near the end of the first wave between December 2019 and April 2020 of the coronavirus disease (COVID-19) outbreak in China [1], and soon the declaration of the COVID-19 pandemic was accompanied by reports of several more clades with different mutations in different countries [2]. Some clades are found to be associated with differences in viral phenotype and immunological response in the patients [3], highlighting the importance of monitoring and assessing emerging variants of SARS-CoV-2.

One of the notable variations, the D614G mutation, encodes a change from aspartic acid to glycine in the C-terminal region of the S1 domain of the viral spike protein of SARS-CoV-2. The detection of the mutant G614 has increased rapidly since February 2020, and G614 had become the dominant variant circulating in most parts of the world by June 2020 [4,5]. Since late 2020, all emerging variants of concern (VOC) have carried the D614G mutation. The rapid spread of G614 suggests it may have a transmission advantage over the wildtype D614 in terms of faster growth rate due to higher reproductive number or shorter generation time or both [6]. This hypothesis is corroborated by in vitro studies which showed that the D614G mutation is correlated with increased infectivity in cell models [7]. However, limited assessment has been conducted to date to quantify the epidemiological fitness of G614 compared with its wildtype predecessor D614 [8,9]. Here we used our previous epidemiological framework for fitness inference of influenza strains [10] to analyse SARS-CoV-2 surveillance and sequence data and characterise the comparative transmissibility of the G614 mutant.

## Methods

### Reconstruction of D614 and G614 phylogeny

For the convenience of mutation analysis, we first downloaded all SARS-CoV-2 sequences submitted on or before 15 June 2020 from GISAID [2], because most circulating SARS-CoV-2 viruses carried G614 after 15 June 2020. Multiple sequence alignments were constructed from the downloaded sequences using MAFFT program, and misalignments at and near the 614th codons were corrected. Then we labelled each sequence with either 'D614' or 'G614' based on the amino acid found at the 614th position in the translated amino acid sequences of the spike gene [4]. We excluded sequences that did not have explicit sample collection dates. In total, 35,377 sequences sampled between 24 December 2019 and 8 June 2020 were used to construct the dataset. A phylogenetic tree was built from these global sequences with high sequencing coverages (i.e. > 50%) of the genomes, using maximum likelihood heuristic search and the GTR + CAT nucleotide substitution model in FastTree v2.1.11 [11].

### Reconstruction of D614 and G614 transmission clusters

We examined the global phylogeny to identify the different local transmission chains of D614 and G614 in each country, for use in the fitness model described below. A strict monophyletic lineage of virus strains from the same country was defined as a local transmission cluster (hereafter 'strict' definition, Supplementary Figure S1). A minimum of two sequences in such a cluster was considered as established local transmission. We included countries with such clusters of D614 and G614 that had cocirculated for a period of at least 2 weeks (i.e. at least two disease generations, assuming a mean generation time of 5–7 days). To avoid potential bias due to stochasticity in sampling, we only included countries with 100 or more sequences during the cocirculation period. We identified 515 D614 clusters and 1,420 G614 clusters among 10,915 sequences in 10 included countries, namely Australia, Belgium, Denmark, Iceland, India, the Netherlands, Portugal, Spain, the United Kingdom (UK) and the United States (US). We also examined the effect of different cut-offs for minimum cluster size (two, three, five, 10 and 20 sequences) in our inference.

### Strict and relaxed definition of D614 and G614 transmission clusters

Compared with human influenza viruses, the SARS-CoV-2 genomes evolved at a relatively slower rate and were intensively sampled, and therefore there were many unresolved polytomic nodes in the phylogeny and identical sequences from different countries [12]. This could potentially break a larger local transmission cluster into multiple smaller ones based on the above-mentioned 'strict' definition. As such, we also considered a 'relaxed' definition under which cluster and non-cluster sequences were grouped into an aggregated cluster if they shared the same parent nodes. See Supplementary Figure S1 for an illustrative example of reconstructed clusters under 'strict' and 'relaxed’ definitions. We evaluated the sensitivity of our fitness estimates to the strict and relaxed definitions, as well as to the inclusion or exclusion of the earliest sequence in each cluster which may represent the potential index case for the cluster and was less likely to be derived from the local sustained transmission chains.

### Constructing the model for estimating the G614 fitness

We assumed that the mutation D614G was the only site of interest that characterised the two cocirculating strains, but their differential transmissibility (if any) might be attributable to the combination of D614G and other mutations. We used D614 and G614 to denote the two strains, and we defined the fitness of G614 (σ) as the ratio of the basic reproduction number of the strain with G614 to the strain with D614: 


$$\sigma =R_{0}^{G}/R_{0}^{D}$$.

We formulated the fitness inference framework under the following assumptions in the base case scenario: (i) both D614 and G614 strains cocirculated locally during the period of fitness estimation; (ii) non-pharmaceutical interventions (NPI) had the same effect on the reproductive number of both strains; (iii) the probability that an infected person is selected for viral sequencing was the same for both strains; (iv) recovery from infection with either strain provided protection against reinfection with both strains during the period of estimation; and (v) the fitness of G614 did not depend on age, and age-specific susceptibility to infection was the same for both strains.

Demographic characteristics such as age and sex are epidemiologically relevant in SARS-CoV-2 transmission and therefore these characteristics divide the population into a number of discrete categories. The next generation matrix (NGM) is often used to calculate the reproductive number: each element in the matrix (*NGM_ij_*) is the number of new infections in category *j* generated by one infection in category *i* within one generation time. Under the base case scenario, the NGM of infections by the G614 strain was *σ* times that of the D614 strain [13]. As the pandemic unfolds, the proportion of G614 infections at time *t*, denoted by 𝜌(*t*), will increase towards 1 if *σ* > 1, remain at the same level if *σ* = 1 and decline towards 0 if *σ* < 1. In our previous work, we have shown that 𝜌(*t*) can be well approximated using the equation:


$$\rho \left(t\right)=\frac{\int_{0}^{t}\sigma g^{G}(t-a)\rho (a)i(a)da}{\int_{0}^{t}\sigma g^{G}(t-a)\rho (a)i(a)da+\int_{0}^{t}g^{D}(t-a)(1-\rho \left(a\right))i(a)da}$$

where *i*(*t*) is the total incidence rate (i.e. including both strains),   *g^D^* and *g^G^*, respectively, are the generation time distribution for D614 and G614 infections (assumed to be gamma distributions with *τ* as the ratio of the mean of *g^G^* to that of *g^D^*). We assumed that *g^D^* had a mean of 5.4 days and standard deviation (SD) 3.8 days (estimated from empirical data [14,15]), and *g^D^* and *g^G^* had the same coefficient of variation. Given that the G614 mutant had displaced the D614 wildtype globally by June 2020, we assumed σ ≥ 1 and τ ≤ 1.

### Effects of importations and introductions

To assess the effects of importations dominated by G614 after late February 2020 for most countries in Europe and the US, we modified the equation for 𝜌(*t*) to include an imported force of infection by G614, which was ϕ_G_ times of the local incidence rate:


$$\rho \left(t\right)=\frac{\int_{0}^{t}(\sigma g^{G}\left(t-a\right)\rho \left(a\right)i\left(a\right)+\varphi_{G}i(a))da}{\int_{0}^{t}(\sigma g^{G}\left(t-a\right)\rho \left(a\right)i\left(a\right)+\varphi_{G}i(a))da+\int_{0}^{t}g^{D}(t-a)(1-\rho \left(a\right))i(a)da}$$

We then estimated ϕ_G_ with other parameters in the inference with the likelihood specified below.

### Data streams and the inference of the G614 fitness

**Figure 1:**
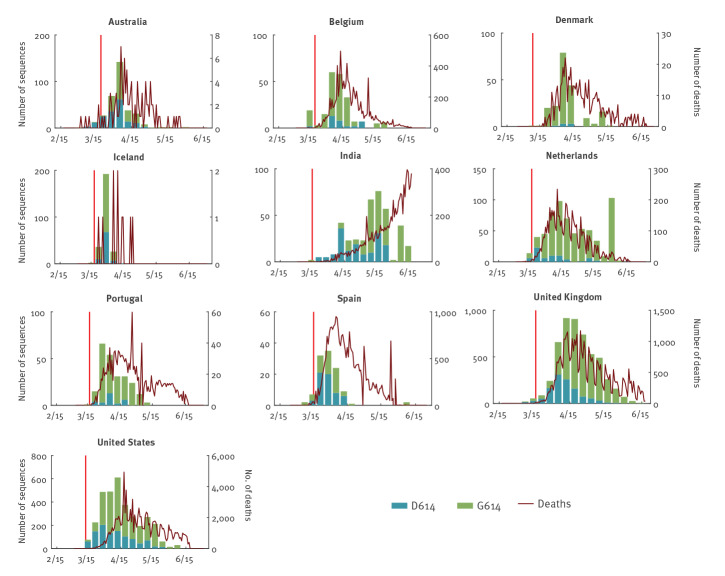
Weekly number of confirmed COVID-19 deaths and of SARS-CoV-2 sequences with D614 and G614, from phylogenetically defined transmission clusters, submitted by 10 included countries, February–June 2020

COVID-19: coronavirus disease; SARS-CoV-2: severe acute respiratory syndrome coronavirus 2.

Our method required two streams of data. The first data stream was the incidence rate *i*(*t*) or its proxy, e.g. using the daily number of confirmed COVID-19 cases or deconvoluting the daily number of COVID-19 deaths with the time between infection and confirmation or death. We denoted this data stream by *ĩ*(*t*).   In the base case scenario, we obtained time series of confirmed COVID-19 deaths from situation updates published by the World Health Organization as the proxies (Figure 1). We assumed that the distribution of the time between infection and death was gamma with mean and SD of 28 and 8.4 days (estimated by integrating the incubation period distribution from Backer et al. [16] and the distribution of the time between symptom onset and death from Verity et al. [17]). We used this distribution to deconvolute the time series of the daily number of deaths to reconstruct an epidemic curve of the daily number of new infections [18]. The second stream was the detections of the D614G mutation, where *Z_d_^D^* and *Z_d_^G^* are the number of SARS-CoV-2 isolates among reconstructed phylogenetic clusters sampled on day   *d* with D614 and G614, respectively (Figure 1).

Clusters with two or more sequences available on GISAID were defined using phylogenetic methods with 'strict' criteria (see Supplementary Figure S1 for an illustrative example of reconstructed clusters under 'strict' and 'relaxed’ definitions). Each cluster stemmed from one or a small number of introductions and at least one transmission chain can be reconstructed from sequences within the same cluster. Only clusters sampled during the cocirculating period of D614 and G614 in each country were included in the analysis. Only countries contributing more than 100 sequences were included in the analysis. The first case of each cluster was included in the analysis. The red lines indicate the date when nationwide travel restrictions from the included country to other countries in the European Union started. The x axis shows date in the format month/day. The SARS-CoV-2 sequences submitted before 8 June 2020 were included in the analysis, specifically sequences submitted between 20 February and 20 May for Australia, 1 March and 20 May for Belgium, 28 February and 9 May for Denmark, 6 and 29 March for Iceland, 3 March and 5 June for India, 1 March and 26 May for the Netherlands, 4 March and 2 May for Portugal, 25 February and 30 May for Spain, 29 January and 2 June for the United Kingdom, and 29 January and 30 May for the United States. The authors, originating and submitting laboratories of the sequences shared via GISAID and used for this analysis are listed in Supplementary Table S3.

We performed a sensitivity analysis of the time between infection and these key events: (i) we assumed the time between infection and sampling was 5, 7, 9 and 12 days with a coefficient of variation of 0.3; (ii) we assumed the time between infection and reporting was 5, 7, 9 and 12 days with a coefficient of variation of 0.3; (iii) we assumed the time between infection and death was 21, 28 and 35 days with a coefficient of variation of 0.3. We used the time series of confirmed COVID-19 cases in the sensitivity analysis because it is more often confounded with temporal fluctuations in reporting rate and testing capacity [19], but our previous simulations had shown that our method is robust against these fluctuations [10].

We did not include China and other East Asian countries in the analysis because no continuous cocirculation was detected in most Asian countries during the study period and there was not enough information from GISAID to avoid misclassifying sequences from imported cases as those from local cases. We substituted *i*(*t*) with *ĩ*(*t*) and the resulting approximate likelihood was


$$L=\prod_{d}\left(\begin{array}{c} Z_{d}^{D}+Z_{d}^{G}\\ Z_{d}^{G} \end{array}\right){\left(\int_{d}^{d+1}\tilde{\rho }(t)dt\right)}^{Z_{d}^{G}}{\left(1-\int_{d}^{d+1}\tilde{\rho }(t)dt\right)}^{Z_{d}^{D}}$$

With this likelihood, the inference was performed in a Bayesian framework with non-informative uniform priors using Markov chain Monte Carlo (MCMC) methods. Three parallel chains were initiated with random starting values of each parameter, and each chain was run with 100,000 iterations. The initial 10,000 samples were discarded as a burn-in phase and the samples were subsequently thinned by 30 to obtain uncorrelated chains. Each MCMC chain was then split in two halves and the Gelman–Rubin algorithm was used to assess convergence of the chain by comparing its two halves.

### Data sharing statement

We collated all data from publicly available data sources. All the information that we used is available in the main text or the supplementary materials.

### Ethical statement

The study was exempt from ethics review by the HKU/HA HKW Institutional Review Board in Hong Kong because only secondary data were collected and analysed in which no human or animal participants were involved.

## Results

### Identification of D614 and G614 cocirculating clusters

**Figure 2:**
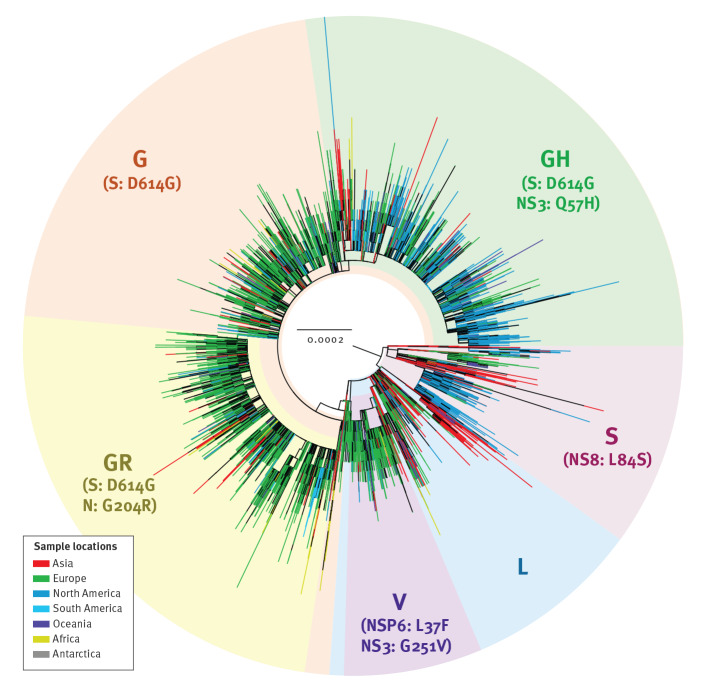
Global phylogeny of SARS-CoV-2, December 2019–June 2020 (n = 26,244)

The global phylogeny of SARS-CoV-2 shows multiple genetic clades and their associated genomic mutations, of which the clade with the G614 mutation is by far the largest (Figure 2). G614 had become dominant in the pandemic in early June 2020 [4], therefore we limited our fitness analysis to sequences collected during the cocirculation period of both strains before 15 June 2020. In the 10 selected countries, the G614:D614 ratio increased over time and the G614 mutant rapidly became dominant (Figure 1).

SARS-CoV-2: severe acute respiratory syndrome coronavirus 2.

The maximum likelihood tree was inferred from the alignment of 26,244 worldwide SARS-CoV-2 genome sequences with high sequencing coverage (i.e. > 50%), using the GTR + CAT substitution model in the FastTree programme. Multiple clades are highlighted with different shading colours, and their associated mutations are indicated in parentheses. Tree tips corresponding to the viral sequences from different continents are annotated in different colours. The authors, originating and submitting laboratories of the sequences shared via GISAID and used for this analysis are listed in Supplementary Table S3.

### Inference of the G614 fitness in transmission

**Figure 3:**
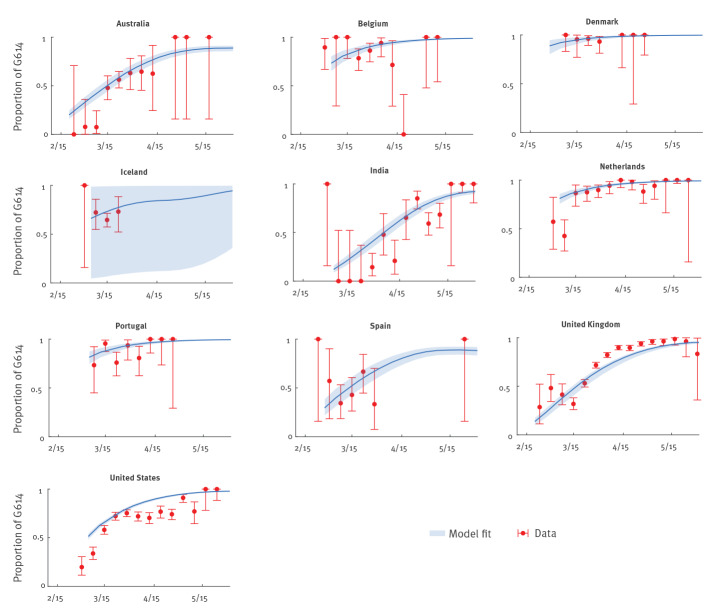
Weekly proportion of SARS-CoV-2 sequences with G614 when both D614 and G614 strains cocirculated, 10 included countries, late January–mid-June 2020 (n = 10,915)

COVID-19: coronavirus disease; SARS-CoV-2: severe acute respiratory syndrome coronavirus 2.

Using confirmed deaths (adjusted for the delay between onset and death) as a proxy for the COVID-19 epidemic curve, we estimated that *σ* was 1.31 (95% credible interval (CrI): 1.28–1.34) and *τ* was 0.99 (95% CrI: 0.96–1.00) across the 10 countries. This means that the basic reproductive number of the G614 mutant was 31% (95% CrI: 28–34) higher than that of the D614 ancestral virus, and the mean generation time of the two strains was essentially the same. The fitted model was congruent with the observed proportions of G614 isolates over time in all 10 countries (Figure 3). If we used confirmed cases instead of confirmed deaths as the proxy for the COVID-19 epidemic curve (Supplementary Figures S2 and S3), then *σ* was 1.23 (95% CrI: 1.19–1.26) and *τ* was 0.96 (95% CrI: 0.90–1.00).

The time series of confirmed COVID-19 deaths was used in the estimation. The red circles and error bars indicate the observed proportion with 95% binomial confidence interval among sequence data. The blue lines and shades indicated the posterior mean and 95% credible interval of the estimates. The authors, originating and submitting laboratories of the sequences shared via GISAID and used for this analysis are listed in Supplementary Table S3.

To assess potential geographical heterogeneity in the transmission advantage of the G614 mutant, we allowed σ to differ among the US, the UK and the remaining locations and reran the inference. The resulting estimates for *σ* were 1.13 (95% CrI: 1.09–1.16), 1.53 (95% CrI: 1.28–1.58) and 1.30 (95% CrI: 1.19–1.42) for the US, the UK and other locations, respectively, with *τ* = 0.99 (95% CrI: 0.93–1.00).

**Figure 4:**
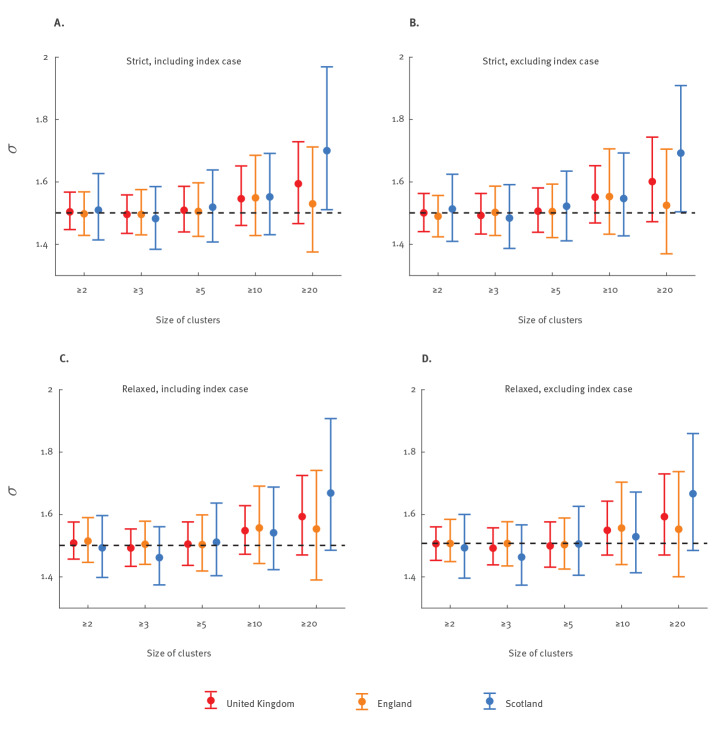
Estimates of SARS-CoV-2 G614 fitness under different phylogenetic definitions and minimum sizes of local transmission clusters, United Kingdom, late January–mid-June 2020 (n = 8,206)

COVID-19: coronavirus disease; SARS-CoV-2: severe acute respiratory syndrome coronavirus 2.

The global phylogeny of SARS-CoV-2 suggested that most countries in Europe (such as the UK [20]) and the US received a large number of importations of G614 since late February 2020. To assess the effects of dominant introductions of G614, we incorporated G614 importation in the fitness estimation by specifically assuming the imported infections consisted of G614 only and the imported G614 force of infection was ϕ_G_ times of the local COVID-19 incidence rate. We performed a sensitivity analysis on sequences from the UK, which is among the countries with the largest number of SARS-CoV-2 genomes made available to the public (Figure 4). The resulting estimate of ϕ_G_ was 0.0012 (0.0010–0.0035), suggesting that the dominant G614 importations were not driving the increase of G614 over time in the UK (Supplementary Figure S4). Similarly, assuming ϕ_G_ was the same in the 10 included countries, the resulting ϕ_G_ estimate was 0.0172 (0.0028–0.0271). See Supplementary Table S1 for estimates of other parameters under this scenario. 

The time series of confirmed COVID-19 deaths was used in the estimation. The circles and error bars indicate the posterior mean and 95% credible intervals of G614 fitness estimates. The horizontal dashed line shows the posterior mean of G614 fitness estimates of the United Kingdom in the base case (including clusters with ≥ 2 sequences in each cluster using the 'strict' cluster definition and assuming that the index case in each cluster was included; it includes clusters reconstructed from England, Scotland, Wales and Northern Ireland). Panels A–D show the base case as in Figures 1 and 3, including clusters with ≥ 2, ≥ 3, ≥ 5, ≥ 10 and ≥ 20 sequences in each cluster using the 'strict' or 'relaxed' cluster definition and assuming that the index case in each cluster was included or excluded. The authors, originating and submitting laboratories of the sequences shared via GISAID and used for this analysis are listed in Supplementary Table S3.

Although G614 introductions occurred later than D614, more clusters with G614 were reconstructed in the 10 included countries and these clusters were larger on average. Since the size of clusters strongly depended on the sampling scheme and sequencing priority in each country, we performed a sensitivity analysis on sequences from the UK to assess the effects of sampling frequency in the G614 fitness estimation. We included only clusters with at least two, three, five, 10 or 20 different patient sequences in the fitness estimation (Figure 4). We found that estimates of *σ* were not sensitive to the minimum cluster sizes up to 20 sequences. The estimations of *σ* were also not sensitive to the definitions of phylogenetic topology (i.e. 'strict' and 'relaxed' definitions; see Methods and Supplementary Figure S1) used to identify the D614 and G614 local transmission clusters (Figure 4).

Although the above results suggested that there was no difference between the generation time of the two strains, we conducted a sensitivity analysis to assess the possibility that the transmission advantage of G614 was entirely due to shorter generation time, i.e. *τ* < 1 and *σ* = 1. The resulting estimate of *τ* was 0.80 (95% CrI: 0.75–0.86), i.e. the mean generation time of G614 was 20% (95% CrI: 14–25) shorter than that of D614. However, this fitted model had significantly higher Akaike information criterion than our base case model, hence supporting our base case conclusion that the mean generation time of the two strains was essentially the same and the transmission advantage of the G614 mutant was entirely due to higher infectivity.

### Effects of G614 fitness in the SARS-CoV-2 transmission dynamics

The inferred value of σ suggested that the herd immunity threshold for the G614 mutant was higher than that for the D614 wildtype. For example, if mixing is homogeneous, the excess is (1−1/*σ*) (1−1/R_0,D614_) where R_0,D614_ is the basic reproductive number of the D614 wildtype. Using the inferred value of *σ* = 1.31, we estimated that the D614G mutation would increase the herd immunity threshold from 50% to 62% (i.e. 12% excess) if R_0,D614_ = 2 and from 67% to 75% (i.e. 8% excess) if R_0,D614_ = 3. More robust estimates of herd immunity threshold would require accounting for heterogeneities in age-dependent physical mixing, susceptibility, infectiousness, etc [21].

Compared with Australia and the US, the countries in Europe experienced earlier introduction of the G614 strain (Table). The proportion of G614 infections reached 19–74% by early March for countries in Europe. Similarly, a more detailed breakdown of the US data showed that the introduction of G614 occurred earlier in the state of New York compared with the state of Washington. Assuming *τ* = 1, we estimated that *σ* was 1.25 (95% CrI: 1.20–1.30) for the state of Washington, but we could not estimate the fitness of G614 for the state of New York because there were no cocirculating clusters of both strains.

**Table:** The proportion of SARS-CoV-2 infections sequences with the G614 mutant when both D614 and G614 started to cocirculate, 10 included countries, late January–early March 2020

Country	GISAID ID of the first sequence in D614 and G614 cocirculating clusters included in the analysis	Sampling date of the first sequence in D614 and G614 cocirculating clusters included in the analysis	𝜌(0) (95% CrI)
Australia	EPI_ISL_420456	22 February 2020	0.132 (0.100–0.169)
Belgium	EPI_ISL_415155	1 March 2020	0.622 (0.528–0.714)
Denmark	EPI_ISL_416143	28 February 2020	0.834 (0.720–0.919)
Iceland	EPI_ISL_427757	6 March 2020	0.501 (0.023–0.975)
India	EPI_ISL_420543	3 March 2020	0.071 (0.050–0.098)
The Netherlands	EPI_ISL_413588	1 March 2020	0.735 (0.665–0.798)
Portugal	EPI_ISL_418011	4 March 2020	0.738 (0.649–0.816)
Spain	EPI_ISL_418251	25 February 2020	0.192 (0.135–0.264)
United Kingdom	EPI_ISL_466615	16 February 2020	0.071 (0.048–0.096)
United States	EPI_ISL_417100	29 February 2020	0.384 (0.349–0.417)

CrI: credible interval; SARS-CoV-2: severe acute respiratory syndrome coronavirus 2.

The authors, originating and submitting laboratories of the sequences shared via GISAID and used for this analysis are listed in Supplementary Table S3.

## Discussion

We have extended a method for estimating antiviral resistance of influenza to estimate the transmission advantage of SARS-CoV-2 mutant variants. Characterising the nonlinear dynamics of the COVID-19 pandemic often requires multiple sources of data and construction of a complex transmission model. Our methods bypass such complexity and are thus easy to implement. In our model, both D614 and G614 viruses co-circulated in the same population during the study period, such that any non-pharmaceutical intervention would have the same effect on the transmissibility of the two viruses. Furthermore, the temporal changes in the non-pharmaceutical interventions were captured by the incidence proxy.

Our findings suggest that the SARS-CoV-2 lineage with the G614 mutation was 31% more transmissible than the ancestral D614 strain. Such increase in fitness allowed the G614 strain to displace the ancestral D614 strain and it became the dominant strain in Europe within 2 months after its first detection. Our findings are consistent with the differential growth rates of D614 and G614 lineages estimated from a different phylodynamic analysis in UK [5]. Our results are also largely consistent with the rate at which COVID-19 was resurging in Beijing in June 2020 in comparison with the spread of the D614-dominated first wave in January and February 2020. Whole genome sequencing showed that the strain causing the June wave in Beijing was genetically closest to the virus isolates in Europe with G614 [8,22]. While 156 local cases were reported between 12 and 31 January for the D614-dominated first wave, 325 local cases were reported between 11 and 30 June for the G614-dominated outbreak. This suggests that the latter was more transmissible given that Beijing had remained extremely vigilant with COVID-19 surveillance and control since mid-January.

We estimated that infection fatality rates were not statistically significant in locations where SARS-COV-2 circulation was dominated by G614, although data were limited (Supplementary Table S2). Although the virus with G614 ostensibly seemed to cause more mild and asymptomatic infections in Beijing’s Xinfadi outbreak, intensive community testing was organised only in June (and thus more mild infections might have been identified) [23]: 96.1% (246/256) of confirmed cases were mild or moderate in June, which was higher than 86.7% (216/249) during the first wave in early 2020 [24]; 7.9% (22/278) of confirmed infections were asymptomatic in June compared with 5.0% (13/262) during the first wave [24].

Our base case results suggest that *R*_0_ of the G614 strain was 1.3 times that of the D614 strain which had been estimated to be 2–2.5 using data from Wuhan, China [25,26]. This is consistent with *R*_0_ estimates of 3–4.5 in Europe and the US where G614 was dominant in mid-2020 [19]. Taken together, these results imply that control measures that were sufficient for controlling D614-dominant outbreaks would only be 70% as effective against G614-dominant outbreaks. For instance, physical distancing interventions were reported to reduce 79% of contacts in Shanghai during the first wave [27], which achieved fast and successful suppression of the first wave by mid-February but may not be sufficient in a situation where *R*_0_ is 3–4.5. Similarly, the critical vaccination coverage (equivalent to the herd immunity threshold) for G614 would be higher than that for D614. 

In the sensitivity analysis, we estimated that *τ* = 0.80 when we assumed *τ* < 1 and *σ* = 1. Thus, an alternative and less probable explanation for the faster doubling time of the G614 strain was that there was no change in *R*_0_ (i.e. *σ* = 1) but the mean generation time of the G614 mutant was around 20% shorter than that of the D614 ancestral virus (i.e. *τ* = 0.80). Using data from the first pandemic wave in the mainland Chinese city Guangzhou, we previously estimated that possibly 44% of all SARS-CoV-2 infection events were presymptomatic transmission and 95% of all transmission would have taken place by day 5 after symptom onset [28]. If the G614 virus were to spread faster but caused slightly milder illness, its current dominance would require a more rapid response (20% faster) in contact tracing and testing to control any outbreak even at the very early stage. However, in this scenario, the critical vaccination coverage for the two strains would be the same because there is no difference in *R*_0_ [29].

Our study had several limitations. Firstly, we only considered the D614G mutation and simply categorised the sequences on GISAID by aligning the spike protein region that contains the locus. We did not consider mutations in other loci that might provide necessary genetic background for D614G and act synergistically to affect the fitness of G614. The mutant D614G was detected sporadically among local cases in the mainland Chinese provinces Guangdong and Zhejiang after February 2020, but no sustained circulation of G614 clusters had been detected in mainland China until the Xinfadi Market outbreak in Beijing in June 2020. The biological mechanism of increased spread of G614 is still unclear. Secondly, we estimated the date of infection approximately by deconvoluting the time series of the dates of sampling for sequence data or the dates of reporting of confirmed cases or deaths. Given the relatively high fitness advantage of G614, the date of exposure or symptom onset should be used instead of the date of sampling to generate more accurate fitness estimates if clinical data of patients could be linked with sequences available on GISAID. Thirdly, our fitness estimation is only applicable when D614 and G614 lineages cocirculate and therefore cannot be used to monitor the fitness of a newly emerged mutant strain that has not yet spread in the community or has already dominated the community transmission. Fourthly, our method compares the relative fitness of two strains. We assumed that other factors that affect SARS-CoV-2 transmission, such as difference in sex and NPI, had the same effects on both strains. Further work is required to consider the differential immune escapes of various VOCs from previous infections or vaccinations, such as the newly emerged variant Omicron. Fifthly, we did not consider a scenario where three or more strains cocirculate and their transmissions might interfere with each other. Although sustained G614 transmission was not detected previously in Guangdong and Zhejiang, the mutant strain may have accumulated several necessary mutations chronologically and exhibited a gradual increase in fitness over time. Categorising all the sequences by D614 and G614 might have oversimplified the biological process and mechanism. Finally, the *σ* estimate from the US seemed to be lower than that of UK and other locations in the Europe. Although we assumed that the two strains cocirculated locally during the study period in the US, our phylogenetic analyses suggested that the spread of D614 and G614 had clear geographical heterogeneity in different US states. Given the limited data availability, we were not able to estimate G614 transmissibility for every individual US state, but more accurate estimates could be obtained for future variants with more SARS-CoV-2 genomic data.

## Conclusion

We have shown that the G614 mutation confers a transmission advantage over the wildtype D614. Monitoring the emergence of mutations and fitness of mutant strains is essential during the COVID-19 pandemic because the spread of mutants can attenuate the effectiveness of outbreak response and control interventions such as development of therapy and vaccines. It is also important to acquire a thorough understanding of viral phenotypes, clinical and epidemiological characteristics of emerging SARS-CoV-2mutants such as D614G, such that surveillance and disease control measures could be adjusted dynamically to counter the evolving risks posed by dominant mutant clades. Although further work is required to adjust for differential immune escapes of various VOC, our method can be readily integrated into the analysis of phylogenetic data in the current SARS-CoV-2 surveillance system, to provide an efficient and timely epidemiological assessment of the transmission potential of emerging mutants.

## Supplementary Data

797000 Supplement

## Supplementary Data

292000 Supplementary_TableS3

## References

[1] Tang X Wu C Li X Song Y Yao X Wu X On the origin and continuing evolution of SARS-CoV-2. Natl Sci Rev. 2020;7(6):1012-23. 10.1093/nsr/nwaa036 34676127 PMC7107875

[2] Global Initiative on Sharing All Influenza Data (GISAID). EpiCoV - Pandemic coronavirus causing COVID-19. Munich: GISAID. [Accessed: 30 Jun 2020] Available from: https://www.gisaid.org

[3] Zhang X Tan Y Ling Y Lu G Liu F Yi Z Viral and host factors related to the clinical outcome of COVID-19. Nature. 2020;583(7816):437-40. 10.1038/s41586-020-2355-0 32434211

[4] Korber B Fischer WM Gnanakaran S Yoon H Theiler J Abfalterer W Tracking changes in SARS-CoV-2 spike: evidence that D614G increases infectivity of the COVID-19 virus. Cell. 2020;182(4):812-827.e19. 10.1016/j.cell.2020.06.043 32697968 PMC7332439

[5] COVID-19 Genomics UK Consortium (COG-UK). Updated analysis of SARS-CoV-2 spike protein variant D614G in the UK: evaluating evidence for effects on transmission and pathogenicity. Cambridge: COG-UK; 2020. Available from: https://www.cogconsortium.uk/wp-content/uploads/2020/07/25th-June-2020-Report-COVID-19-Genomics-UK-COG-UK-Consortium.pdf

[6] Wallinga J Lipsitch M . How generation intervals shape the relationship between growth rates and reproductive numbers. Proc Biol Sci. 2007;274(1609):599-604. 10.1098/rspb.2006.3754 17476782 PMC1766383

[7] Li Q Wu J Nie J Zhang L Hao H Liu S The impact of mutations in SARS-CoV-2 spike on viral infectivity and antigenicity. Cell. 2020;182(5):1284-1294.e9. 10.1016/j.cell.2020.07.012 32730807 PMC7366990

[8] Volz E Hill V McCrone JT Price A Jorgensen D O’Toole Á Evaluating the effects of SARS-CoV-2 Spike mutation D614G on transmissibility and pathogenicity. Cell. 2021;184(1):64-75.e11. 10.1016/j.cell.2020.11.020 33275900 PMC7674007

[9] Volz EM, Hill V, McCrone JT, Price A, Jorgensen D, O'Toole A, et al. Evaluating the effects of SARS-CoV-2 spike mutation D614G on transmissibility and pathogenicity. medRxiv. 2020.07.31.20166082. Preprint. https://doi.org/10.1101/2020.07.31.20166082 10.1016/j.cell.2020.11.020 PMC7674007 33275900

[10] Leung K Lipsitch M Yuen KY Wu JT . Monitoring the fitness of antiviral-resistant influenza strains during an epidemic: a mathematical modelling study. Lancet Infect Dis. 2017;17(3):339-47. 10.1016/S1473-3099(16)30465-0 27914853 PMC5470942

[11] Price MN Dehal PS Arkin AP . FastTree 2--approximately maximum-likelihood trees for large alignments. PLoS One. 2010;5(3):e9490. 10.1371/journal.pone.0009490 20224823 PMC2835736

[12] Lam TT-Y . Tracking the genomic footprints of SARS-CoV-2 transmission. Trends Genet. 2020;36(8):544-6. 10.1016/j.tig.2020.05.009 32527617 PMC7253973

[13] Diekmann O Heesterbeek JA Roberts MG . The construction of next-generation matrices for compartmental epidemic models. J R Soc Interface. 2010;7(47):873-85. 10.1098/rsif.2009.0386 19892718 PMC2871801

[14] Leung K Wu JT Liu D Leung GM . First-wave COVID-19 transmissibility and severity in China outside Hubei after control measures, and second-wave scenario planning: a modelling impact assessment. Lancet. 2020;395(10233):1382-93. 10.1016/S0140-6736(20)30746-7 32277878 PMC7195331

[15] Kwok KO Wong VWY Wei WI Wong SYS Tang JW-T . Epidemiological characteristics of the first 53 laboratory-confirmed cases of COVID-19 epidemic in Hong Kong, 13 February 2020. Euro Surveill. 2020;25(16):2000155. 10.2807/1560-7917.ES.2020.25.16.2000155 32347198 PMC7189647

[16] Backer JA Klinkenberg D Wallinga J . Incubation period of 2019 novel coronavirus (2019-nCoV) infections among travellers from Wuhan, China, 20-28 January 2020. Euro Surveill. 2020;25(5):2000062. 10.2807/1560-7917.ES.2020.25.5.2000062 32046819 PMC7014672

[17] Verity R Okell LC Dorigatti I Winskill P Whittaker C Imai N Estimates of the severity of coronavirus disease 2019: a model-based analysis. Lancet Infect Dis. 2020;20(6):669-77. 10.1016/S1473-3099(20)30243-7 32240634 PMC7158570

[18] Leung K Wu JT Xu K Wein LM . No detectable surge in SARS-CoV-2 transmission attributable to the April 7, 2020 Wisconsin election. Am J Public Health. 2020;110(8):1169-70. 10.2105/AJPH.2020.305770 32552029 PMC7349432

[19] Flaxman S Mishra S Gandy A Unwin HJT Mellan TA Coupland H Estimating the effects of non-pharmaceutical interventions on COVID-19 in Europe. Nature. 2020;584(7820):257-61. 10.1038/s41586-020-2405-7 32512579

[20] COVID-19 Genomics UK (COG-UK) Consortium. SARS-CoV-2 genomic epidemiology in the UK. . Cambridge: COG-UK; 2020. Available from: https://www.cogconsortium.uk/wp-content/uploads/2020/06/28th-May-2020-Report-COVID-19-Genomics-UK-COG-UK-Consortium.pdf

[21] Britton T Ball F Trapman P . A mathematical model reveals the influence of population heterogeneity on herd immunity to SARS-CoV-2. Science. 2020;369(6505):846-9. 10.1126/science.abc6810 32576668 PMC7331793

[22] Tan W, Niu P, Zhao X, Pan Y, Zhang Y, Chen L, et al. Notes from the field: Reemergent cases of COVID-19—Xinfadi wholesales market, Beijing Municipality, China, June 11, 2020. China CDC Weekly. 2020:1-3. Available from: http://weekly.chinacdc.cn/en/article/doi/10.46234/ccdcw2020.132 10.46234/ccdcw2020.132 PMC8428450 34594688

[23] Chinese Center for Disease Control and Prevention (CCDC). Situation updates of Beijing's COVID-19 outbreak in June 2020. Beijing: CCDC; 2020. Available from: http://www.chinacdc.cn/yw_9324/202006/P020200626557038667020.pdf

[24] Tian S Hu N Lou J Chen K Kang X Xiang Z Characteristics of COVID-19 infection in Beijing. J Infect. 2020;80(4):401-6. 10.1016/j.jinf.2020.02.018 32112886 PMC7102527

[25] Wu JT Leung K Bushman M Kishore N Niehus R de Salazar PM Estimating clinical severity of COVID-19 from the transmission dynamics in Wuhan, China. Nat Med. 2020;26(4):506-10. 10.1038/s41591-020-0822-7 32284616 PMC7094929

[26] Kucharski AJ Russell TW Diamond C Liu Y Edmunds J Funk S Early dynamics of transmission and control of COVID-19: a mathematical modelling study. Lancet Infect Dis. 2020;20(5):553-8. 10.1016/S1473-3099(20)30144-4 32171059 PMC7158569

[27] Zhang J Litvinova M Liang Y Wang Y Wang W Zhao S Changes in contact patterns shape the dynamics of the COVID-19 outbreak in China. Science. 2020;368(6498):1481-6. 10.1126/science.abb8001 32350060 PMC7199529

[28] He X Lau EHY Wu P Deng X Wang J Hao X Temporal dynamics in viral shedding and transmissibility of COVID-19. Nat Med. 2020;26(5):672-5. 10.1038/s41591-020-0869-5 32296168

[29] Keeling MJ, Rohani P. Modeling infectious diseases in humans and animals. Princeton: Princeton University Press; 2011.

