# Supplementary material for: Estimating the transmission advantage of the D614G mutant strain of SARS-CoV-2, December 2019 to June 2020
Source: Euro Surveill. 2021 Dec 9;26(49):2002005. doi: 10.2807/1560-7917.ES.2021.26.49.2002005 (PMC8662801; doi:10.2807/1560-7917.ES.2021.26.49.2002005)
Supplement: Supplement [file 20-02005_WU_Supplement.pdf]

This supplementary material is hosted by Eurosurveillance as supporting information alongside the article ‘Estimating the transmission advantage of the D614G mutant strain of SARS-CoV-2, December 2019 to June 2020', on behalf of the authors, who remain responsible for the accuracy and appropriateness of the content. The same standards for ethics, copyright, attributions and permissions as for the article apply. Supplements are not edited by Eurosurveillance and the journal is not responsible for the maintenance of any links or email addresses provided therein.

Supplementary information

The infection fatality risks in locations predominated by D614 or G614.

To test the hypothesis that the G614 mutation might affect the clinical severity of SARS-CoV-2 infection, we estimated the infection fatality risks (IFRs) as the ratio of laboratory-confirmed deaths to the estimated number of infections in locations or settings with SARS-COV-2 circulation predominated by D614 or G614. The daily number of confirmed deaths were obtained from the websites of local public health agencies. For locations where extensive contact tracing and testing had been conducted, the number of infections were estimated as the number of reported infections; for locations where seroprevalence studies had been conducted, the number of infections were estimated by the product of the seroprevalence and the population size accordingly. We assumed on average it takes 21 days for infected individuals to develop consistently detectable antibodies after infection [1], and the time between infection and deaths is 28 days [2].

Among locations where SARS-COV-2 circulation was dominated by D614, we estimated that IFR ranged from 0.90% (0.75-1.06) in mainland Chinese provinces outside Hubei to 1.0% (0.40-2.04) among passengers from the Princess Diamond Cruise (Table 2). The IFR estimates were lower but not significantly different among locations where SARS-COV-2 circulation was dominated by G614, ranging from 0.43% (0.37-0.56) in Geneva, Switzerland to 0.83% (0.65-1.10) in New York City, US (Supplementary Table S2).

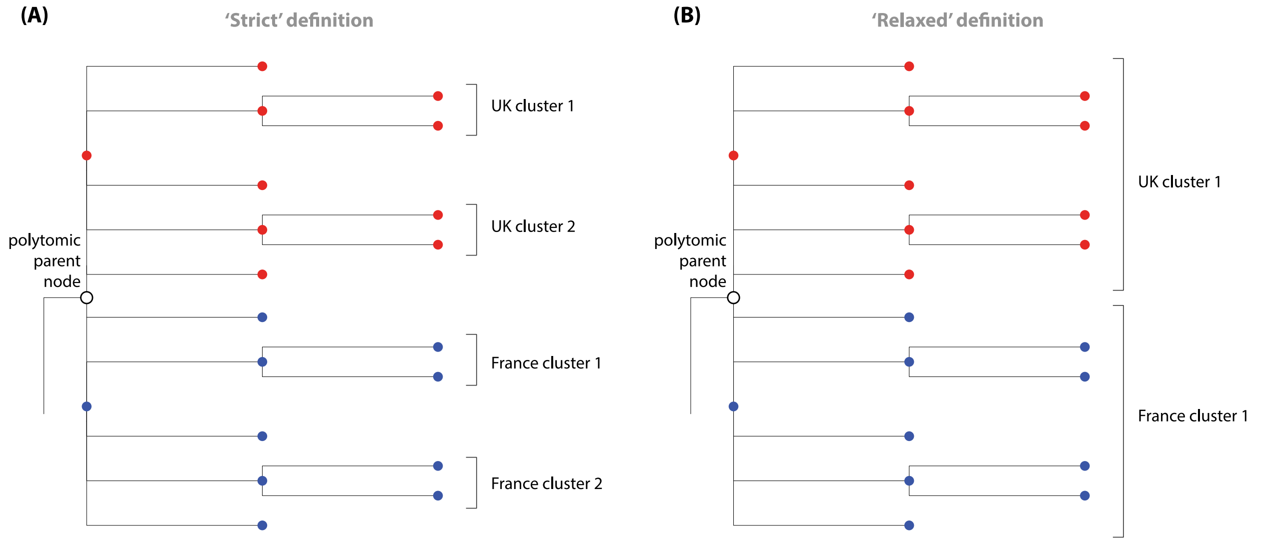

Supplementary Figure S1. Illustration of 'strict' and 'relaxed' definitions of transmission clusters reconstructed with phylogenetic methods. Solid circles in red and blue colors are sequences from UK and France respectively. In 'strict' definition, only multiple sequences from the same country sharing strictly monophyletic relationship are considered as a transmission cluster. In 'relaxed' definition, cluster and non-cluster sequences of the same country are aggregated into a larger cluster if they share the same parent node (e.g. the open circle in the tree) even if it is a polytomy and consists of child nodes from different countries.

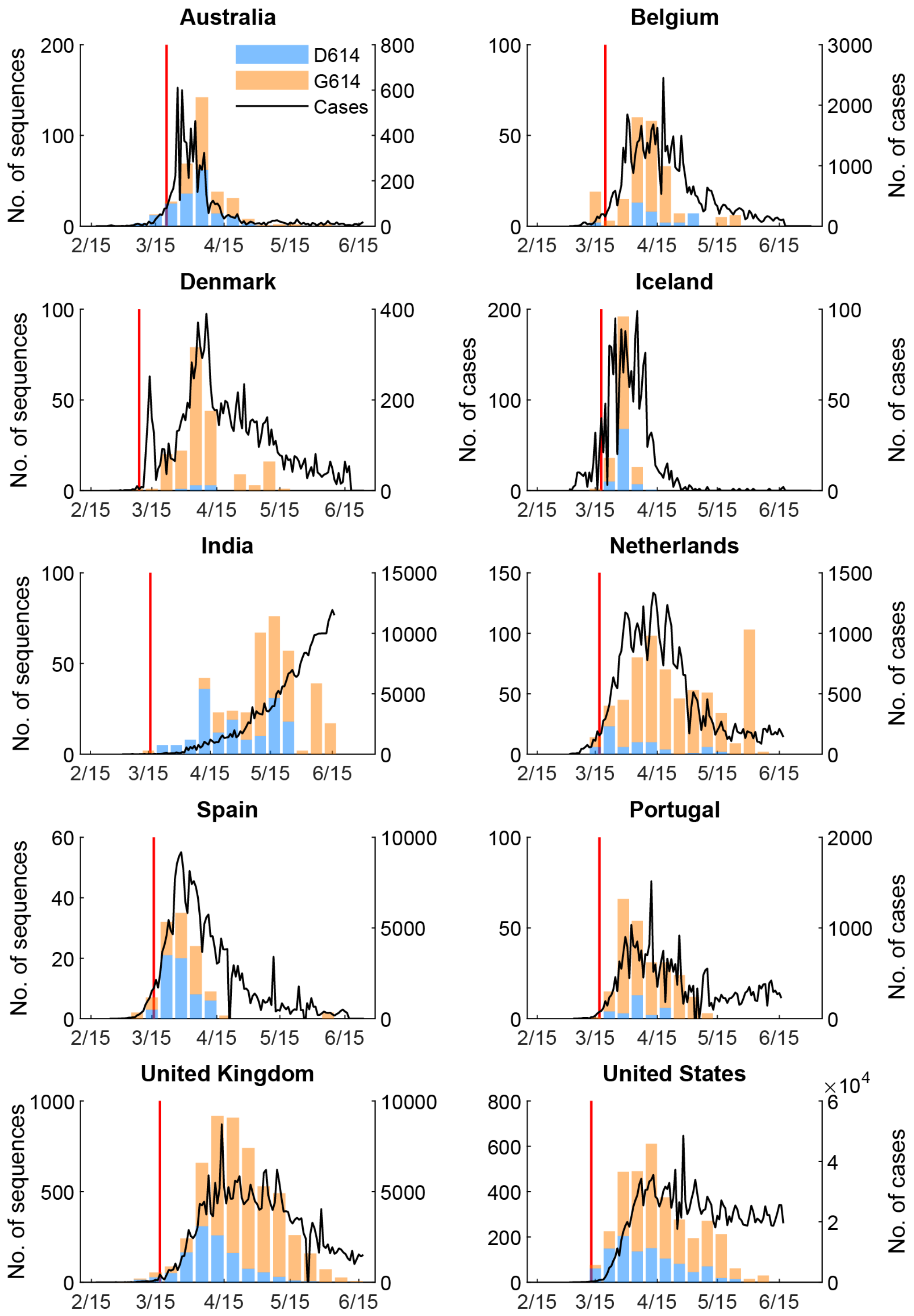

Australia
Belgium
Denmark
Iceland
India
Netherlands
Spain
Portugal
United Kingdom
United States
D614
G614
Cases
No. of sequences
No. of cases
2/15
3/15
4/15
5/15
6/15
×10^4

Supplementary Figure S2. Weekly number of confirmed COVID-19 cases and the weekly number of D614 and G614 sequences from clusters with two or more cases, submitted by Australia, Belgium, Denmark, Iceland, India, Netherlands, Spain, Portugal, UK and US. Clusters with two or more sequences on GISAID were defined by phylogenetic methods with "strict" criteria. Each cluster stems from one or a small number of introductions and at least one transmission chain can be reconstructed from sequences within the same cluster. Only clusters sampled during the co-circulating period of D614 and G614 strains in each country were included in the analysis. Only countries with more than 100 sequences from at least 5 co-circulating clusters were included in the analysis. The red lines indicated the date when major travel restriction from or to countries of European Union started.

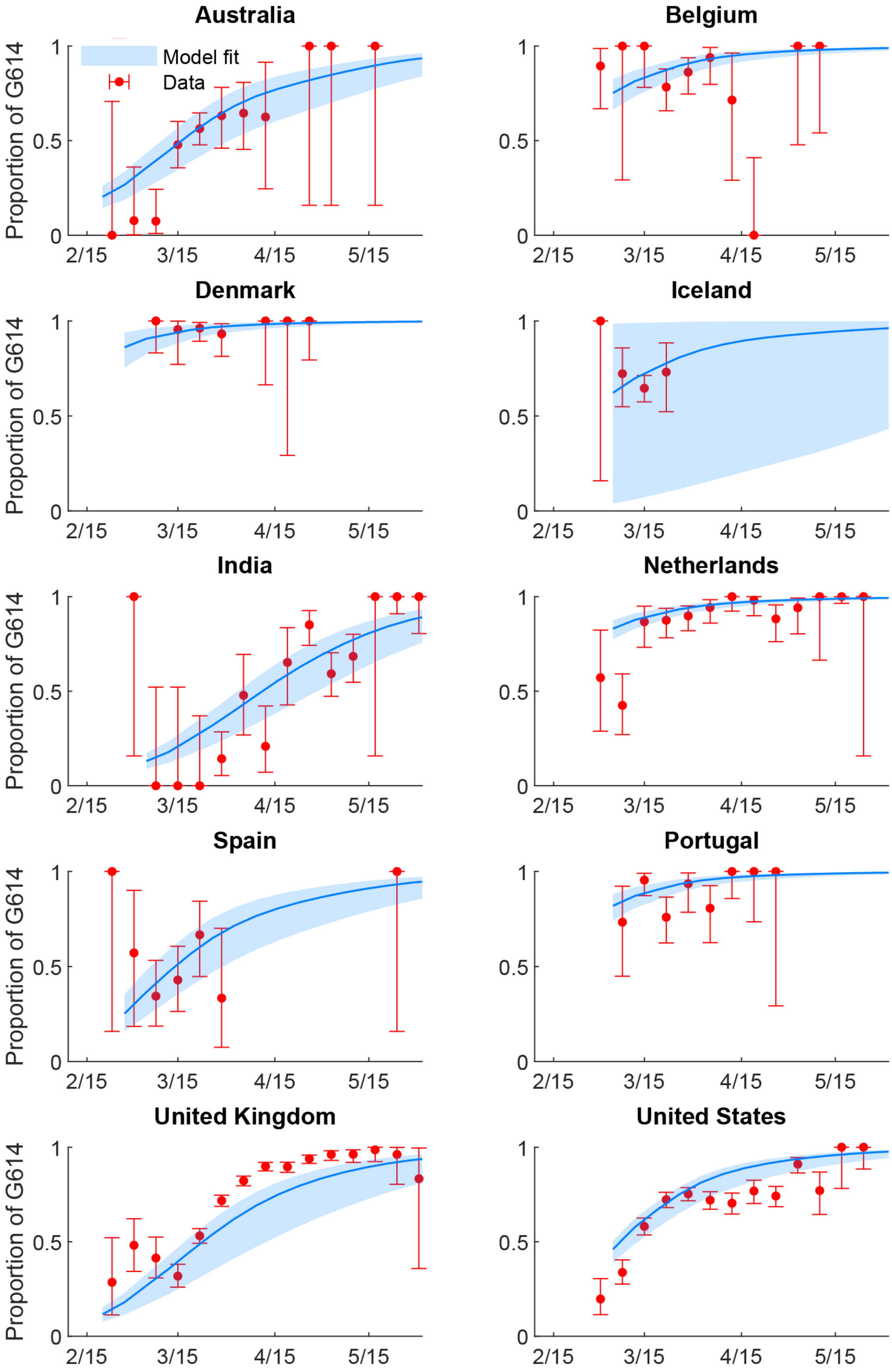
Australia
Belgium
Denmark
Iceland
India
Netherlands
Spain
Portugal
United Kingdom
United States
Model fit
Data
Proportion of G614
0
0.5
1
2/15
3/15
4/15
5/15

Supplementary Figure S3. The weekly proportion of G614 infections between late January and early May when both D614 and G614 strains cocirculated. The time series of confirmed COVID-19 cases was used in the estimation. The red circles and error bars indicated the observed proportion with 95% binomial CIs among sequence data. The blue lines and shades indicated the posterior mean and 95% CrI of the estimates.

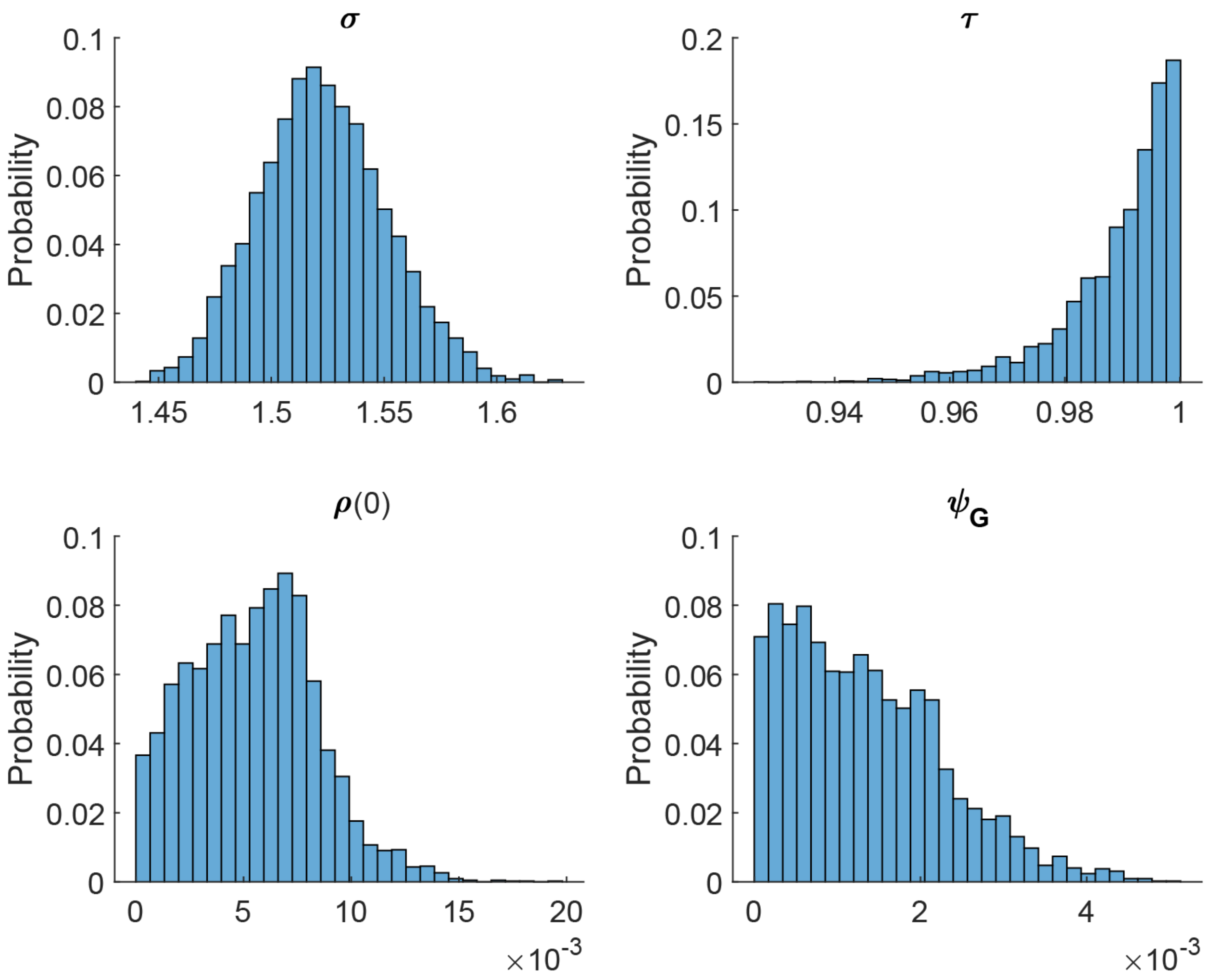

Supplementary Figure S4. The posterior distribution of parameters if the force of infection of G614 importations is included in the fitness estimation in the UK. The base case includes clusters with ≥2 sequences in each cluster using the "strict" cluster definition assuming index case in each cluster was included. Clusters were reconstructed from sequences sampled from England, Scotland, Wales and Northern Ireland.

Supplementary Table S1. The posterior distribution of parameters if the force of infection of G614 importations is included in the fitness estimation in the ten selected countries

Parameters	Country	Posterior mean (95% CrI)
The ratio of the basic reproduction number of the G614 strain to that of the D614 strain σ		1.273 (1.233-1.313)
The ratio of the mean generation time of the G614 strain to that of the D614 strain τ		0.993 (0.961-1.000)
The proportion of G614 infections when both D614 and G614 started to cocirculate $\rho(0)$*	Australia	0.099 (0.063-0.145)
	Belgium	0.627 (0.529-0.717)
	Denmark	0.841 (0.724-0.926)
	Iceland	0.497 (0.022-0.978)
	India	0.023 (0.002-0.068)
	Netherlands	0.745 (0.675-0.810)
	Spain	0.173 (0.106-0.253)
	Portugal	0.745 (0.656-0.821)
	United Kingdom	0.021 (0.001-0.068)
	United States	0.386 (0.351-0.417)
The scaling factor of the force of infections of G614 importations φ_G		0.017 (0.003-0.027)

* Sampling dates of the 1st sequence in D614 and G614 cocirculating clusters included in the analysis of each country are the same as Table 1.

Supplementary Table S2. Estimated infection fatality risks in countries or regions with SARS-COV-2 circulation predominated by D614 or G614

Country/Region/Setting	Circulation period of interest	Strain	No. of confirmed deaths	No. of infections or seroprevalence (95% CI)	IFR (95% CI)	Method/ Source
Wuhan, Hubei Province, China	Jan-Mar 2020	D614	3,869	To et al<br>3.76% (2.21-5.95)<br>Xu et al<br>3.67% (2.71-4.86)	 0.93% (0.59-1.58)<br><br>0.95% (0.72-1.29)	[3-5]
Provinces outside Hubei in mainland China	Jan-Mar 2020	D614	135	15,076*	0.90% (0.75-1.06)	[2]
Diamond Princess Cruise	Feb 2020	D614	7	705	0.99% (0.40-2.04)	[6]
Washington State, US	Feb-Apr 2020	D614 & G614	610<br>(as of 8 Apr §)	1.13% (0.70-1.94)	0.71% (0.41-1.14)	[7]
New York City, New York State, US	Feb-Apr 2020	G614	13,495<br>(as of 26 Apr §)	19.3% (14.6-24.9)	0.83% (0.65-1.10)	[8, 9]
Geneva, Switzerland	Feb-May 2020	G614	230<br>(as of 16 May §)	10.8% (8.2-12.3)	0.43% (0.37-0.56)	[10]
London, UK	Feb-May 2020	D614 & G614	7,957<br>(as of 15 May §)	17.5% (13.4-22.8)	0.51% (0.39-0.67)	[11]

* Assuming the number of reported local cases was close to the number of local infections because the ascertainment rates were ~100% in provinces outside Hubei given the intensive and proactive case finding

§ 7 days after the last week of the last estimate of seroprevalence, assuming it takes 21 days on average to develop consistently detectable antibodies after infection and the time between infection and deaths is 28 days

References

1. Perera RA, Mok CK, Tsang OT, Lv H, Ko RL, Wu NC, et al. Serological assays for severe acute respiratory syndrome coronavirus 2 (SARS-CoV-2), March 2020. Eurosurveillance. 2020;25(16):2000421.
2. Leung K, Wu JT, Liu D, Leung GM. First-wave COVID-19 transmissibility and severity in China outside Hubei after control measures, and second-wave scenario planning: a modelling impact assessment. The Lancet. 2020.
3. Chinese Center for Disease Control and Prevention. Dashboard of reported 2019-nCoV cases 2020 [Available from: http://2019ncov.chinacdc.cn/2019-nCoV/.
4. To KK-W, Cheng VC-C, Cai J-P, Chan K-H, Chen L-L, Wong L-H, et al. Seroprevalence of SARS-CoV-2 in Hong Kong and in residents evacuated from Hubei province, China: a multicohort study. The Lancet Microbe. 2020.
5. Xu X, Sun J, Nie S, Li H, Kong Y, Liang M, et al. Seroprevalence of immunoglobulin M and G antibodies against SARS-CoV-2 in China. Nature Medicine. 2020:1-3.
6. Russell TW, Hellewell J, Jarvis CI, Van Zandvoort K, Abbott S, Ratnayake R, et al. Estimating the infection and case fatality ratio for coronavirus disease (COVID-19) using age-adjusted data from the outbreak on the Diamond Princess cruise ship, February 2020. Eurosurveillance. 2020;25(12):2000256.
7. Havers FP, Reed C, Lim T, Montgomery JM, Klena JD, Hall AJ, et al. Seroprevalence of antibodies to SARS-CoV-2 in 10 sites in the United States, March 23-May 12, 2020. JAMA internal medicine. 2020;180(12):1576-86.
8. Stadlbauer D, Tan J, Jiang K, Hernandez MM, Fabre S, Amanat F, et al. Repeated cross-sectional sero-monitoring of SARS-CoV-2 in New York City. Nature. 2020:1-5.
9. Stadlbauer D, Tan J, Jiang K, Hernandez M, Fabre S, Amanat F, et al. Seroconversion of a city: Longitudinal monitoring of SARS-CoV-2 seroprevalence in New York City. medRxiv. 2020:2020.06.28.20142190.
10. Stringhini S, Wisniak A, Piumatti G, Azman AS, Lauer SA, Baysson H, et al. Seroprevalence of anti-SARS-CoV-2 IgG antibodies in Geneva, Switzerland (SEROCoV-POP): a population-based study. The Lancet. 2020.
11. Public Health England. Sero-surveillance of COVID-19 2020 [Available from: https://www.gov.uk/government/publications/national-covid-19-surveillance-reports/sero-surveillance-of-covid-19.
